# Phylogenetic Analysis of the SQUAMOSA Promoter-Binding Protein-Like Genes in Four *Ipomoea* Species and Expression Profiling of the *IbSPLs* During Storage Root Development in Sweet Potato (*Ipomoea batatas*)

**DOI:** 10.3389/fpls.2021.801061

**Published:** 2022-01-21

**Authors:** Haoyun Sun, Jingzhao Mei, Weiwei Zhao, Wenqian Hou, Yang Zhang, Tao Xu, Shaoyuan Wu, Lei Zhang

**Affiliations:** ^1^Jiangsu Key Laboratory of Phylogenomics and Comparative Genomics, School of Life Sciences, Jiangsu Normal University, Xuzhou, China; ^2^Department of Biochemistry and Molecular Biology, 2011 Collaborative Innovation Center of Tianjin for Medical Epigenetics, Tianjin Key Laboratory of Medical Epigenetics, Key Laboratory of Immune Microenvironment and Disease (Ministry of Education), School of Basic Medical Sciences, Tianjin Medical University, Tianjin, China

**Keywords:** *Ipomoea*, SPL transcription factor, evolutionary patterns, root development, expression profiles

## Abstract

As a major plant-specific transcription factor family, *SPL* genes play a crucial role in plant growth, development, and stress tolerance. The SPL transcription factor family has been widely studied in various plant species; however, systematic studies on *SPL* genes in the genus *Ipomoea* are lacking. Here, we identified a total of 29, 27, 26, and 23 *SPLs* in *Ipomoea batatas*, *Ipomoea trifida*, *Ipomoea triloba*, and *Ipomoea nil*, respectively. Based on the phylogenetic analysis of SPL proteins from model plants, the *Ipomoea* SPLs were classified into eight clades, which included conserved gene structures, domain organizations and motif compositions. Moreover, segmental duplication, which is derived from the *Ipomoea* lineage-specific whole-genome triplication event, was speculated to have a predominant role in *Ipomoea SPL* expansion. Particularly, tandem duplication was primarily responsible for the expansion of SPL subclades IV-b and IV-c. Furthermore, 25 interspecific orthologous groups were identified in *Ipomoea*, rice, *Arabidopsis*, and tomato. These findings support the expansion of *SPLs* in *Ipomoea* genus, with most of the *SPLs* being evolutionarily conserved. Of the 105 *Ipomoea SPLs*, 69 were predicted to be the targets of miR156, with seven *IbSPLs* being further verified as targets using degradome-seq data. Using transcriptomic data from aboveground and underground sweet potato tissues, *IbSPLs* showed diverse expression patterns, including seven highly expressed *IbSPLs* in the underground tissues. Furthermore, the expression of 11 *IbSPLs* was validated using qRT-PCR, and two (*IbSPL17/IbSPL28*) showed significantly increased expression during root development. Additionally, the qRT-PCR analysis revealed that six *IbSPLs* were strongly induced in the roots under phytohormone treatments, particularly zeatin and abscisic acid. Finally, the transcriptomic data of storage roots from 88 sweet potato accessions were used for weighted gene co-expression network analysis, which revealed four *IbSPLs* (*IbSPL16/IbSPL17/IbSPL21/IbSPL28*) clusters with genes involved in “regulation of root morphogenesis,” “cell division,” “cytoskeleton organization,” and “plant-type cell wall organization or biogenesis,” indicating their potential role in storage root development. This study not only provides novel insights into the evolutionary and functional divergence of the *SPLs* in the genus *Ipomoea* but also lays a foundation for further elucidation of the potential functional roles of *IbSPLs* on storage root development.

## Introduction

The SQUAMOSA promoter-binding protein-like (*SPL*) genes are of the plant-specific transcription factor families, which play fundamental roles in plant growth, development, and stress tolerance (Guo et al., 2008; Chen et al., 2010; Preston and Hileman, 2013; Chen et al., 2015; Wang and Wang, 2015). The *SPL* genes predominantly contain the SQUAMOSA promoter-binding (SBP) domain, which comprises three distinct motifs: two non-interleaved zinc-binding sites (Cys-Cys-Cys-His and Cys-Cys-His-Cys) and one nuclear localization signal (NLS) at the C-terminus (Guo et al., 2008). *SPL* genes (*AmSBP1* and *AmSBP2*) were first discovered in snapdragon (*Antirrhinum majus*), following their role in flower development (Klein et al., 1996). Since then, numerous homologs have been identified and characterized in model species, such as *Arabidopsis thaliana* (Cardon et al., 1999), rice (*Oryza sativa*) (Xie et al., 2006), and tomato (*Solanum lycopersicum*) (Salinas et al., 2012). With the increasing number of sequenced genomes, *SPL* members have been increasingly annotated and reported in non-model plants, such as apple (*Malus domestica*) (Li et al., 2013), *Jatropha curcas* (Yu et al., 2020), pepper (*Capsicum annuum*) (Zhang et al., 2016), poplar (*Populus trichocarpa*) (Li and Lu, 2014), and soybean (*Glycine max*) (Tripathi et al., 2017).

SPL genes in model plants have been well studied, showing functional divergence. In *A. thaliana*, AtSPL3, AtSPL9, and AtSPL10 have been reported to regulate root development (Yu et al., 2015; Barrera-Rojas et al., 2020). Similar roles have also been reported in rice (Shao et al., 2019) and apple (Xu et al., 2017). Various studies have further found that SPL proteins participate in vegetative and reproductive phase transitions, for example, AtSPL3/4/5 promotes flowering by directly inducing AP1, FUL and LFY expression, which are flowering integrator genes (Yamaguchi et al., 2009). Additionally, SPL proteins are involved in fruit development and grain yield. For example, LeSPL-CNR regulates cell wall disassembly and carotenoid biosynthesis during fruit ripening in tomato (Orfila et al., 2002; Manning et al., 2006); *OsSPL16* expression promotes cell division and grain filling, with positive results in grain width and yield in rice (Wang et al., 2015). *SPL* genes are also considered as miR156 targets, thus forming a functional miR156-SPL regulatory network (Wang and Wang, 2015). In the miR156-SPL network, AtSPL9 negatively affects anthocyanin accumulation (Gou et al., 2011), whereas OsSPL7 enhances disease resistance against bacterial blight (Liu et al., 2019). The functions of *SPL* genes have been comprehensively studied in *Arabidopsis* and other model plants; however, their functionality in *Ipomoea* are relatively scarce.

The genus *Ipomoea*, which includes 500–600 species, possesses the largest number of species in the family *Convolvulaceae* (Austin and Huáman, 1996). *Ipomoea* species are widely and globally distributed with great value in the fields of industry and agriculture (Austin and Huáman, 1996; Liu, 2017; Morita and Hoshino, 2018). For example, Japanese morning glory, *Ipomoea nil* (L.) Roth. (2n = 2x = 30), is cultivated as an ornamental plant due to its diverse flower color patterns (Morita and Hoshino, 2018). Sweet potato, *Ipomoea batatas* (L.) Lam. (2n = 6x = 90), is ranked as the seventh most important crop globally due to its strong adaptability, stable yields, and high nutritional value (Liu, 2017). The storage roots of sweet potato, which are mainly harvested, have significant nutrient content and yield (Zhang et al., 2020). The initiation and development of storage roots is known as a complex and genetically programmed process (Ravi et al., 2014). Although several studies have reported the formation and development of storage root at the morphological, physiological, and molecular level (Nakatani, 1991; Wang et al., 2005; Tanaka et al., 2008; Noh et al., 2010; Dong et al., 2019; Huan et al., 2020), the underlying mechanisms of storage root development have not yet been fully elucidated. Up to now, the genomes of four species (*I. batatas, Ipomoea trifida*, *Ipomoea triloba*, and *I. nil*) have been sequenced in *Ipomoea* (Hoshino et al., 2016; Yang et al., 2017; Wu et al., 2018). Among these species, *I. trifida* is the most closely related diploid to *I. batatas*, followed by *I. triloba* and *I. nil* (Wu et al., 2018). The reported haplotype-resolved genome assembly of *I. batatas* is of low-quality, making it difficult to accurately identify and characterize genes. Contrastingly, the genome assembly of the other three diploid relatives is of high-quality and can be used as robust references for *I. batatas*. Therefore, genome availability makes it possible to perform a genome-wide comparative analysis of *SPL* genes in *Ipomoea*.

In this study, genome-wide identification and characterization of *SPL* genes were performed in four publicly available *Ipomoea* species, including *I. batatas*, *I. trifida*, *I. triloba*, and *I. nil*. Following this, the phylogenetic relationships and evolutionary patterns of *SPL* genes were investigated in these four species. *IbSPL* gene expression patterns were determined using transcriptome and qRT-PCR in different organs or under various hormone treatments. Finally, weighted gene co-expression network analysis (WGCNA) was used to construct the co-expression network and infer the putative functions for the *IbSPL* genes in the storage root of sweet potato. This work not only provides insights into the evolutionary conservation and diversification of *SPL* genes in the genus *Ipomoea* but also lays the foundation for further research on *IbSPL* genes related to storage root development in sweet potato.

## Materials and Methods

### Identification of SQUAMOSA Promoter-Binding Protein-Like Genes in *I. nil, I. triloba, I. trifida, and I. batatas*

The genomes of four *Ipomoea* species (including *I. nil, I. triloba*, *I. trifida*, and *I. batatas*) were downloaded from the ‘‘*Ipomoea nil* Genome Project^1^ “ (Hoshino et al., 2016), ‘‘Sweetpotato Genomic Resource^2^ “ (Wu et al., 2018), and ‘‘Sweet potato genome browser^3^ “ (Yang et al., 2017), respectively. *SPL* genes in these four species were identified using the following three methods. First, the 16 *A. thaliana* SPL proteins^4^ were used as queries to find *SPL* homologs using BLASTP program with a threshold of *e*-value < 1*e*-3. Second, the Hidden Markov Model (HMM) of the SBP (PF03110) domain was downloaded from the Pfam database (El-Gebali et al., 2019) and used to identify putative SPL proteins using the hmmsearch (El-Gebali et al., 2019) program. Third, all the candidate SPL proteins obtained from the BLASTP and hmmsearch analysis were submitted to the SMART (Letunic and Bork, 2018) and ScanProsite databases (de Castro et al., 2006) to confirm an SBP domain presence. Proteins lacking the SBP domain were excluded, while the remaining were considered as the SPL proteins. Additionally, a manual examination was performed on the structures of identified *IbSPLs* to correct genome assembly errors.

The BUSCA online software (Savojardo et al., 2018) was used to predict the subcellular localization of *Ipomoea* SPL proteins. An in-house Perl script was used to analyze the physical and chemical properties of *Ipomoea* SPL proteins, such as protein length, molecular weight (MW, kD) and isoelectric point (pI).

### Phylogenetic Analysis of SQUAMOSA Promoter-Binding Protein-Like Proteins

Phylogenetic analysis was performed on the SPL proteins from *Chlamydomonas reinhardtii*, *A. thaliana*, *J. curcas*, *M. domestica*, *O. sativa*, *P. trichocarpa*, *S. lycopersicum*, and the four *Ipomoea* species using the following steps: first, MAFFT software (v7.45) (Katoh and Standley, 2013) was used to obtain full-length SPL proteins’ multiple alignments; second, Gblocks program (v0.71b) (Castresana, 2000) was used to select conserved blocks from the multiple alignments; third, MEGA X software (Kumar et al., 2018) was used to construct a neighbor-joining phylogenetic tree with 1000 bootstrap replications, with the CRR1 protein from *C. reinhardtii* was set as an outgroup; finally, Evolview website was used to visualize the tree (Subramanian et al., 2019). Similarly for the *Ipomoea* SPL proteins, a neighbor-joining phylogenetic tree was constructed using the aforementioned.

### Analysis of the Gene Structure, Protein Domain, and Motif

The exon/intron positions of all *Ipomoea SPL* genes were obtained from the downloaded GFF3 files of the genomic database. The domain organizations of *Ipomoea* SPL proteins were annotated based on the SMART database results (Letunic and Bork, 2018). The conserved sequences in each domain were shaded at four levels using GeneDoc. The motif compositions of *Ipomoea* SPL proteins were analyzed through MEME online database (Bailey et al., 2009), with the maximum number set to 10. Finally, the gene structure, domain organization, and motif composition were drawn using Tbtools (Chen et al., 2020).

### Gene Duplication, Orthology, and Selection Analysis

MCScanX software (Wang et al., 2012) was used to identify collinear blocks within or between species to classify the *SPL* genes into five different types: singleton, dispersed, proximal, tandem, and segmental duplication. The synteny relationships of the collinearity blocks in each *Ipomoea* species were visualized using Circos (Krzywinski et al., 2009). OrthoMCL software (Li et al., 2003) was used to detect orthologous groups among the diverse *SPL* genes. For each orthologous gene pair, *Ks* (synonymous substitution rate), *Ka* (non-synonymous substitution rate), and *Ka/Ks* ratio (evolutionary constraint) were calculated using PAML (Yang, 2007).

### Prediction of miR156-Targeted Genes

Publicly available datasets were used to identify miR156 sequences in *Ipomoea*. A total of 58 miRNA transcriptomes deposited in National Center for Biotechnology Information (NCBI) (Coordinators, 2018) (16 in PRJNA471495, 2 in PRJNA474012, 11 in PRJNA592001, 12 in PRJNA599544, 12 in PRJNA600587, and 5 in PRJNA638516) were collected (Supplementary Table 5; Kuo et al., 2019; Saminathan et al., 2019; Yang et al., 2020; Liu et al., 2021). Trimmomatic software (version 0.39) (Bolger et al., 2014) was used to filter the raw miRNA sequencing data, which removed low-quality reads and sequencing adaptors. Finally, using *I. batatas* as the reference genome, the miRDeep2 (version 1.1.4) pipeline (Kuang et al., 2019) was employed to identify miR156 sequences with default parameters.

For the *Ipomoea SPL* genes, miR156 target sites were predicted using the psRNATarget server (Dai et al., 2018) with default settings. The predicted miR156-SPL interactions in *I. batatas* were validated using five degradomes (one in PRJNA592001 and four in PRJNA600587) (Yang et al., 2020; Liu et al., 2021) downloaded from public databases (Supplementary Table 5). After filtering out the low-quality reads and sequencing adaptors, the CleaveLand4 pipeline (Addo-Quaye et al., 2009) was used to identify miR156 cleavage sites. The identified targets with categories 0–3 and *p*-values < 0.05 were considered to be reliable miR156 target genes.

### Promoter Analysis of *Ipomoea* SQUAMOSA Promoter-Binding Protein-Like Genes

The 2000 bp sequence upstream of the start codon for all *Ipomoea SPL* genes was retrieved using an in-house Perl script and submitted to the PlantCARE program (Lescot et al., 2002) to predict *cis*-acting elements as previously described (Chen et al., 2019). The distribution of *cis*-acting elements in each promoter was determined using TBtools (Chen et al., 2020).

### Plant Materials and Hormone Treatments

Sweet potato (*I. batatas* cv. Xuyu34) plants used in this study were provided by the Xuzhou Academy of Agricultural Sciences, Xuzhou, Jiangsu, China. According to institutional, national, and international guidelines, these samples do not require specific permissions for research purposes. The plants were grown in greenhouses on the campus of Jiangsu Normal University, Xuzhou, China. For organ-specific expression analysis, the tissues of young leaves, mature leaves, flowers, and roots (10, 20, 40, 60, 80, 90, and 100 DAT roots with 0.3, 2, 7, 25, 37, 52, and 60 mm in diameter, respectively) were collected. For hormone treatments, stems with 4–5 leaves were cut and planted in 1/8 Hoagland solution to initiate adventitious root development for 10 days. Then stem cuttings with similar growth conditions were chosen and planted in 1/8 Hoagland solution separately containing 100 μM abscisic acid (ABA), indole-3-acetic acid (IAA), zeatin (ZT), and methyl-Jasmonate (MeJA). Stem cuttings without any hormone treatment were set as a control. Adventitious roots from the stem cuttings were collected at 0, 6, 12, 24, and 48 h post the treatments. Three biological replicates were collected for each sample. All samples were frozen in liquid nitrogen and finally stored at –80°C for subsequent use.

### RNA Extraction and qRT-PCR Analysis

For analyzing expression patterns of *IbSPL* genes in different tissues or under phytohormone treatment, total RNA for each sample was extracted using the RNApure Plant Kit (CWBio, Beijing, China), following the manufacturers’ instructions. For investigating the miR156-SPL interactions, total RNA was extracted using TRIzol reagent (Invitrogen, CA, United States) according to the manufacturers’ instructions. The first cDNA strand was synthesized from 1.0 μg total pure RNA using the HiFiScript cDNA Synthesis Kit (CWBio, Beijing, China). The reverse transcription primer and qRT-PCR primer for miR156 were designed as previous study described (Zhou et al., 2020). Gene-specific primers for each *IbSPL* gene were designed using primer3 (Untergasser et al., 2012). qRT-PCR was performed *via* the Bio-rad CFX Connect™ Real-Time System (Bio-Rad, CA, United States) using 2 × Q3 SYBR qPCR Master Mix (Universal) premix (Tolo Biotechnology, Shanghai, China). *IbARF* gene was used as a reference gene for normalizing the expression levels (Park et al., 2012). The relative transcript abundance for each gene was calculated with mean ± SD of biological triplicate samples using the 2^–ΔΔ*CT*^ approach (Livak and Schmittgen, 2001). The primers used are listed in Supplementary Table 10.

### Analysis of the Expression Patterns of *IbSPLs* Using Published Transcriptomic Data

To explore tissue- and developmental stage-specific expression patterns of *IbSPL* genes, publicly available transcriptome datasets from two previous studies (Ding et al., 2017; Wu et al., 2018; Supplementary Table 8) were used: one included eight different tissues from cultivar Xuzi3 and Yan252 under the BioProject accession number PRJCA000640 in National Genomics Data Center (NGDC) (National Genomics Data Center and Partners, 2020), and the other included eight different stages during root development from cultivar Beauregard under the BioProject accession number PRJNA491292 in NCBI (Coordinators, 2018). Transcriptome analysis was performed as described in our previous study (Zhang et al., 2020). The downloaded raw fastq files were filtered using Trimmomatic (version 0.39) (Bolger et al., 2014), and then were mapped to sweet potato genome Taizhong6 (Yang et al., 2017) using STAR (version 2.7.1a) software under the 2-pass mapping mode (Dobin et al., 2013). RSEM (Li and Dewey, 2011) was used to calculate Fragments Per Kilobase of transcript per Million mapped reads (FPKM) values for each gene. Finally, a heatmap was plotted based on the normalized expression values of 29 *IbSPL* genes using the pheatmap package in R.

### Construction of Co-expression Networks Involving *IbSPL* and Other *I. batatas* Genes in Sweet Potato Storage Root

Transcriptomic datasets of mature storage roots of 88 sweet potato accessions were obtained from a previous study (Supplementary Table 11; Ding et al., 2017) under the BioProject accession number PRJCA000642 in NGDC (National Genomics Data Center and Partners, 2020). Weighted co-expression network construction and module detection were performed using the R package WGCNA (version 1.4.9) (Langfelder and Horvath, 2008) with the following parameters: power = 9, minModuleSize = 30, cutHeight = 0.25, and network module export weight threshold = 0.05. The sub-network was subsequently visualized using Cytoscape (Smoot et al., 2011). *eggNOG-mapper* (version 2) (Huerta-Cepas et al., 2017) was used to assign the functional annotation to sweet potato genes, and Clusterprofiler (Yu et al., 2012) was used to perform GO enrichment analysis for genes co-expressed with *IbSPLs* (adjusted *P*-value < 0.05).

### Statistical Analysis

The qRT-PCR results were analyzed using ANOVA (one-way analysis of variance) followed by LSD test. Statistically significant differences at *p* < 0.05 are indicated using different letters.

## Results

### Identification of SQUAMOSA Promoter-Binding Protein-Like Genes in Four *Ipomoea* Species

**FIGURE 1:**
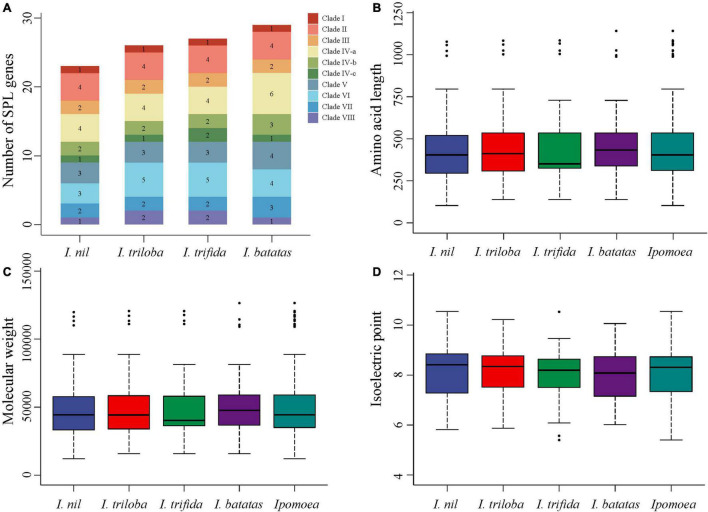
Comparison of *SPL* genes in four *Ipomoea* species. **(A)** Comparison of the number and ratio of *SPL* genes in four *Ipomoea* species, with different colors representing each clade. **(B–D)** Comparison of SPL protein lengths, MWs, and pIs in four *Ipomoea* species, respectively.

BLASTP and HMM were used to identify the *SPL* genes in *Ipomoea* species, while SMART and ScanProsite were used to validate the results. A total of 29, 27, 26, and 23 *SPL* genes were identified in *I. batatas* (*Ib*), *I. trifida* (*Itf*), *I. triloba* (*Itb*), and *I. nil* (*In*), respectively. The *Ipomoea SPL* genes were renamed according to their chromosomal location (Supplementary Table 1). The numbers of *SPL* genes and their total percentage in each species are displayed in Figure 1A. The results showed that the *I. nil* genome had the least number of *SPL* genes compared to the other three species.

Subcellular localization analysis showed the nuclear localization of most *Ipomoea* SPL proteins (94, 89.52%) (Supplementary Table 1), suggesting their critical role in regulatory functions. Furthermore, the physical and chemical properties of SPL proteins were significantly differed within species but exhibited similar patterns among the four species (Figures 1B–D and Supplementary Table 1). Moreover, amino acid numbers in the *Ipomoea* SPL proteins ranged from 103 (*InSPL9*) to 1141 (*IbSPL11*), the MWs varied between 12.01 (*InSPL9*) and 126.51 (*IbSPL11*) kDa, and the pIs ranged from 5.40 (*ItfSPL14*) to 10.55 (*InSPL15*).

### Comparative Phylogenetic Analysis of *Ipomoea* SQUAMOSA Promoter-Binding Protein-Like Genes

**FIGURE 2:**
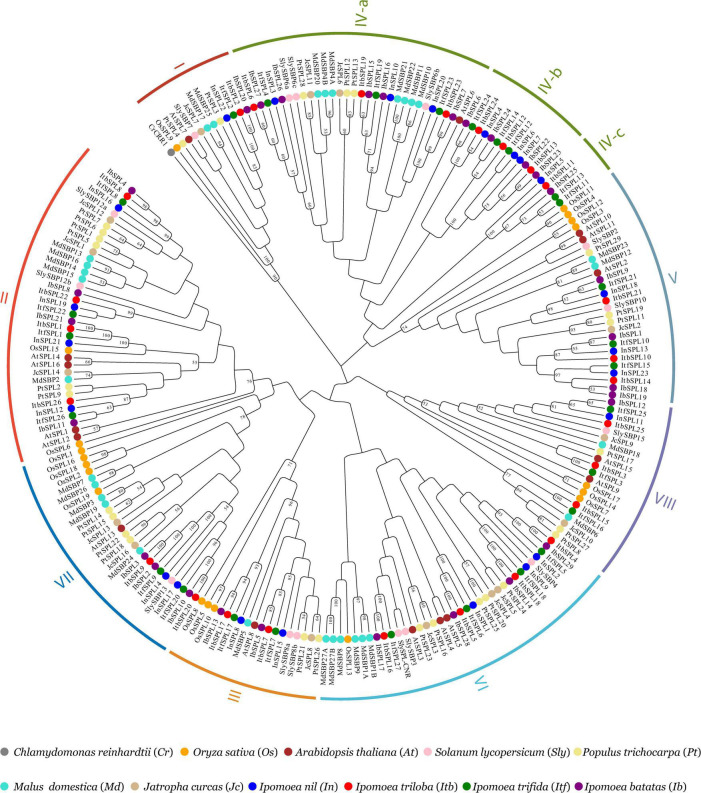
Phylogenetic analysis of SPL proteins from *I. batatas*, *I. trifida*, *I. triloba*, *I. nil*, *C. reinhardtii*, *A. thaliana*, *J. curcas*, *M. domestica*, *P. trichocarpa*, *S. lycopersicum*, and *O. sativa.* The rooted phylogenetic tree was constructed based on the conserved domain of 229 SPLs using the neighbor-joining method with 1000 bootstrap replications. The CrCRR1 protein from *C. reinhardtii* was used as an outgroup to root the tree. Numbers on the tree indicate bootstrap support (values <50% not shown). Each colored arcs indicates the different clades of the SPLs. SPL members from the same species are marked with the same colors: Blue, *I. nil*; red, *I. triloba*; green, *I. trifida*; purple, *I. batatas*; gray, *C. reinhardtii*; orange, *O. sativa*; brown, *A. thaliana*; pink, *S. lycopersicum*; tan, *J. curcas*; turquoise, *M. domestica*; khaki, *P. trichocarpa*.

The evolutionary relationship between the *Ipomoea SPL* genes was explored *via* a rooted neighbor-joining phylogenetic tree, which was constructed using 105 SPL proteins from four *Ipomoea* species and 124 SPL proteins from seven other plant species (*A. thaliana*, *J. curcas*, *M. domestica*, *O. sativa*, *P. trichocarpa*, *S. lycopersicum*, and *Chlamydomonas reinhardii*) (Figure 2 and Supplementary Table 2). Based on the classification of SPLs from *A. thaliana, S. lycopersicum*, and *O. sativa* (Cardon et al., 1999; Xie et al., 2006; Salinas et al., 2012), *Ipomoea SPL* genes were classified into eight clades (I–VIII) (Figures 1, 2). All clades had at least one *SPL* gene in each *Ipomoea* species, indicating *SPL* conservation across *Ipomoea* genomes. However, the number of *SPLs* in certain clades was highly variable among *Ipomoea* species, suggesting a diversity of *SPLs* in the genus *Ipomoea*. Clade I was the smallest subfamily, containing only one member for each *Ipomoea* species while clade IV had the highest number of *SPLs* (>26%) in genus *Ipomoea*, with further divisions into three subclades: IV-a, IV-b, and IV-c. Members of the IV-b and IV-c subclades only comprised *SPL* genes from the *Ipomoea* species and no homologs of other species, indicating that the *Ipomoea SPL* genes in these two subclades were evolutionary conserved. Additionally, the phylogenetic analysis also indicated that most *IbSPL* genes were closer to *ItfSPL* genes than either *ItbSPL* or *InSPL* genes, supporting the fact that *I. trifida* is the most closely related diploid to hexaploid sweet potato (Wu et al., 2018). Moreover, the number of *SPL* genes in *Ipomoea* species (the average number of *SPL* genes in the four *Ipomoea* species was 26) greatly increased by approximately 2 times compared to that in *S. lycopersicum* (13), respectively. These results indicate the extensive expansion of *Ipomoea SPL* genes after the speciation of *S. lycopersicum*.

### Gene and Protein Structure of the *Ipomoea* SQUAMOSA Promoter-Binding Protein-Like Family

The structural diversity of the *Ipomoea SPL* genes was explored using intron/exon structure analysis (Supplementary Figure 1). Gene structure illustrations showed a high variation in the number of exons, ranging from 2 to 15 (Supplementary Figure 1a and Supplementary Table 1). For example, *IbSPL20* contained the highest exons (15), whereas most *Ipomoea SPL* genes in Clade VI contained the least exons (2). Moreover, most *Ipomoea SPL* genes in the same clade exhibited similar gene structures, despite belonging to different species. *SPL* gene in clades I and II contained the highest exons, ranging from 10 to 15, while *SPL* genes in the remaining clades had 2–7 exons (except *IbSPL19*). These results suggested that the gain or loss of exon/intron had occurred during the *Ipomoea SPL* gene evolution, resulting in their functional divergence.

*Ipomoea* SPL protein features were investigated by analyzing the conserved domains using multiple sequence alignment. The results showed that the SPL members had the SBP domain, which comprised two non-interleaved zinc finger-like structures (Zn-1/2) and one NLS motif (Supplementary Figures 1c, 2). Based on the alignments of the *Ipomoea* SPLs, the Zn-2 motif showed higher conservation than the Zn-1 and NLS motifs, which was consistent with the results in *Rosaceae* (Jiang et al., 2021) and *Oryza* species (Zhong et al., 2019; Supplementary Figure 2). The Zn-2 motif in all *Ipomoea* SPLs was a Cys-Cys-His-Cys (C2HC) type (except IbSPL23) whereas the Zn-1 motif showed varied types: Cys-Cys-Cys-Cys (C4) type in clade I and Cys-Cys-Cys-His (C3H) type in the remaining clades. Moreover, other conserved domains were identified in specific clades. For instance, SPLs in clade I and II possessed a DEXDc domain (Supplementary Figure 3), which is involved in ATP-dependent DNA unwinding (Caruthers and McKay, 2002); SPLs in clade II contained Ankyrin repeats (Supplementary Figure 4), which are considered to be significant for mediating protein-protein interactions (Li et al., 2006).

To gain a better understanding of *Ipomoea* SPL protein characteristics, the MEME software was used to explore the motif compositions (Supplementary Figures 1d, 5). The results showed that *Ipomoea* SPL proteins within the same clade showed similar motif compositions, while those in different clades exhibited distinct variations in motif composition. In brief, all *Ipomoea* SPL proteins had two motifs (motif 2 and 3), which were a part of the SBP domain (Supplementary Figure 2); clade I and II had four motifs (motif 4, 5, 6, and 7), with motif 4 being the DEXDc domain (Supplementary Figure 3);clade II had motif 8, which consisted of Ankyrin repeats (Supplementary Figure 4); clade IV had motif 10, which had unknown functions.

### Gene Duplication, Orthology Relationship, and Selective Pressure of *Ipomoea* SQUAMOSA Promoter-Binding Protein-Like Genes

**FIGURE 3:**
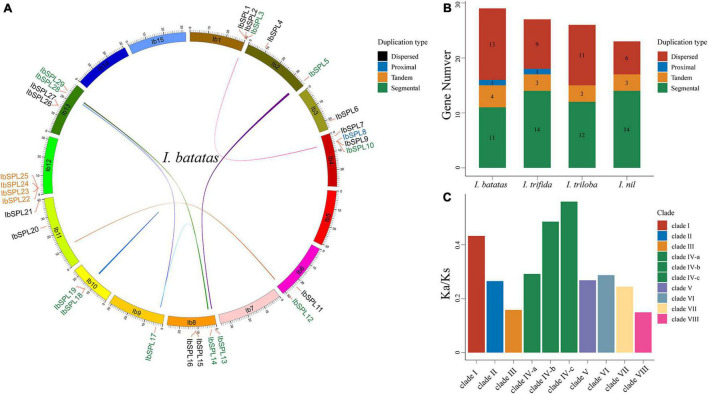
Syntenic relationships and selective pressure of *Ipomoea* SPLs. **(A)** The collinear relationship of *SPL* genes in *I. batatas*, wherein the colored lines indicate syntenic regions. *IbSPL* genes are classified into four duplicated types, each indicated by a different color. **(B)** The distribution of *SPLs* duplication modes in four *Ipomoea* species. **(C)** The mean *Ka/Ks* values in each clade.

To investigate the gene duplication modes of *Ipomoea SPL* genes, MCScanX (Wang et al., 2012) was used to perform gene collinearity analysis in each *Ipomoea* species. All *Ipomoea SPL* genes were estimated to be duplicated (absence of singleton mode), with segmental (51, 48.57%) mode as the dominant mode compared to the other duplication modes: dispersed (39, 37.14%), tandem (13, 12.38%), and proximal (2, 1.90%) (Figures 3A,B; Supplementary Table 1; Supplementary Figure 6). These results indicate that segmental duplication has played a predominant role in the evolution and expansion of *Ipomoea SPL* genes. Additionally, tandem duplication was found to be the predominant model in the IV-b and IV-c subclades, suggesting the expansion of *SPL* genes in these two clades *via* tandem duplication.

The orthologous relationships among the *SPL* genes were determined using OrthoMCL (Li et al., 2003) across *O. sativa*, *A. thaliana*, *S. lycopersicum*, and the four *Ipomoea species*. A total of 25 (1, 2, 2, 8, 3, 5, 2, and 2) orthologous groups in the eight clades (clade I to VIII) were identified, respectively (Supplementary Table 3). Among these groups, nine groups had genes originating from *O. sativa*, suggesting that these *SPL* genes may have originated prior to the split of monocots and dicots; four groups had genes from *A. thaliana* but were absent in *O. sativa*, implying that they originated after the divergence of monocots and dicots; ten groups had genes that existed only in the genus *Ipomoea*, indicating their origination *via* a common ancestor of the *Ipomoea* lineage. Furthermore, the potential functions of certain *Ipomoea SPL* genes could be inferred from their orthologs in *O. sativa*, *A. thaliana*, and *S. lycopersicum*.

To understand the divergence of *Ipomoea SPL* genes, the *Ka*, *Ks*, and *Ka/Ks* ratios for all orthologous groups were calculated using PAML software (Yang, 2007; Figure 3C; Supplementary Table 4). As a result, the mean *Ka/Ks* values of all clades were lower than 1.0, suggesting the evolution of *Ipomoea SPL* genes under the pressure of purifying selection. Genes in clade VIII showed the lowest mean *Ka/Ks* values (0.15) compared to those in the other clades, indicating their evolution under strong positive selection. Contrastingly, genes in subclades IV-b and IV-c exhibited the highest *Ka/Ks* values, implying that these two subclades have generally diverged much more rapidly than the other clades.

### miR156 Target Site of *Ipomoea* SQUAMOSA Promoter-Binding Protein-Like Genes

**FIGURE 4:**
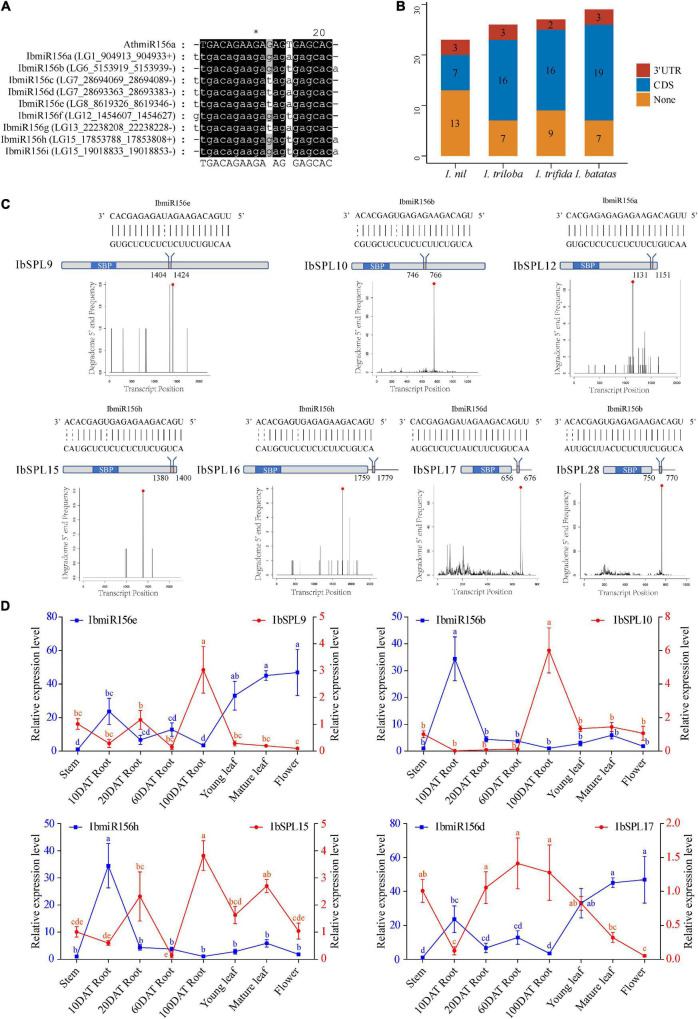
miR156 target site of the *Ipomoea SPL* genes. **(A)** Multiple alignments of identified *IbmiR156* sequences. **(B)** The summary of miR156-targeted *SPL* genes in the *Ipomoea* genus. **(C)** Schematic diagram of *IbSPL* genes targeted by *IbmiR156*. The top diagram represents the complementary sequences between *IbmiR156* and their targets. The gray box indicates the CDS region, the blue box represents the SBP domain, and the red box indicates the *IbmiR156* target site. The diagram below is the target plots (t-plots) of *IbmiR156* targets confirmed by degradome sequencing. **(D)** Expression correlation between *IbmiR156* and *IbSPLs*. The lines represent the abundance of *IbmiR156* and *IbSPLs* in different tissues. The Y-axis on the left and right indicates the relative expression levels of the *IbmiR156* and *IbSPLs*, respectively. Relative expression was calculated using the 2^–ΔΔ*CT*^ method. The error bars indicate the standard deviations of the three biological replicates. Different letters indicate statistically significant differences at *p* < 0.05.

A total of nine IbmiR156 members were identified in *I. batatas* (Figure 4A) using the publicly available miRNA transcriptomes (Supplementary Table 5). To explore the roles of miR156-mediated post-transcriptional regulation of *SPLs* in the genus *Ipomoea*, the transcripts of all the 105 *Ipomoea SPL* genes were searched for the target site of miR156 using psRNATarget (Dai et al., 2018). As a result, a total of 69 *SPL* genes were found to be potential miR156 targets, including 10 *InSPLs*, 19 *ItbSPLs*, 18 *ItfSPLs*, and 22 *IbSPLs* (Figure 4B and Supplementary Table 1). Among the miR156 target *SPL* genes, most of which (84%) the sites recognized by miR156 were located downstream of the SBP domain in the CDS region, then followed by the 3′-UTR (Figure 4B).

The predicted miR156-SPL interactions in *I. batatas* were validated using publicly available degradomes (Supplementary Table 5). The results showed that seven miR156-SPL interactions predicted by psRNATarget were confirmed by degradome sequencing (Figure 4C and Supplementary Table 1). Notably, the miR156 target sites of *IbSPL9*, *IbSPL10*, *IbSPL12*, and *IbSPL15* were located in the CDS region, while the target sites of *IbSPL16*, *IbSPL17*, and *IbSPL28* were located in the 3′-UTR region. The four IbmiR156-IbSPL pairs (IbmiR156b-IbSPL10, IbmiR156d-IbSPL17, IbmiR156e-IbSPL9, and IbmiR156h-IbSPL15) validated through degradomes data, were further selected for expression analysis by qRT-PCR. To understand the regulatory mechanisms of selected miR156 genes, correlation in the expression pattern of miR156 and their target *SPLs* was determined in different tissues (Figure 4D). The expression pattern of IbmiR156e was higher in flower, followed by mature leaf, young leaf, 10DAT root, 60DAT root, 20DAT root, and stem; conversely, the opposite trend was observed for its target IbSPL9. The expression of IbmiR156d and IbSPL17 also showed negative correlation in different tissues. While the expression of IbmiR156b-IbSPL10 and IbmiR156h-IbSPL15 were partially negatively correlated in some tissues.

### *Cis*-Acting Elements in the Promoters of *Ipomoea* SQUAMOSA Promoter-Binding Protein-Like Genes

To understand the regulatory mechanisms and potential functions of *Ipomoea SPL* genes, *cis*-acting elements were analyzed in the 2000 bp upstream sequence from the start codon for all *SPL* genes by using the PlantCARE database (Lescot et al., 2002). A total of 4088 putative *cis*-acting elements were identified and divided into four categories: light responsiveness, plant growth, phytohormone, and abiotic/biotic stress response (Supplementary Tables 6, 7). As shown in Supplementary Figure 7a, *SPL* genes in the same clades exhibited similar *cis*-acting element compositions in the promoter, indicating their conserved biological functions. Among these categories, the abiotic/biotic stress response category covered the largest portion (43.66%), followed by the light response (25.93%), phytohormone response (20.77%), and plant growth (9.64%) categories (Supplementary Figure 7b). In the abiotic/biotic stress response category, MYB/MYC (responds to abiotic stress signals), STRE (metal-responsive element), WUN-motif (wound-responsive element), and LTR (low-temperature-responsive element) elements were found. In the light responsiveness category, Box 4, G-box, GT1-motif, TCT-motif, GATA-motif, and MRE elements were found, with the Box 4 motif as the most common (24%) element. As for the phytohormone response category, the ABRE element (responds to ABA), CGTCA-motif (responds to MeJA), ERE (responds to ethylene), TCA-element (responds to salicylic acid), and as-1 (responds to auxin) were commonly found, appearing in more than 60 *Ipomeoa SPL* genes. In the plant growth category, ARE elements essential for the anaerobic induction, CAT-box related to meristem expression and O2-site involved in zein metabolism regulation were the three major elements. Therefore, analysis of the *cis*-acting elements suggested that *Ipomoea SPL* genes participate in various biological processes.

### Expression Profiles of *IbSPL* Genes in Different Tissues

**FIGURE 5:**
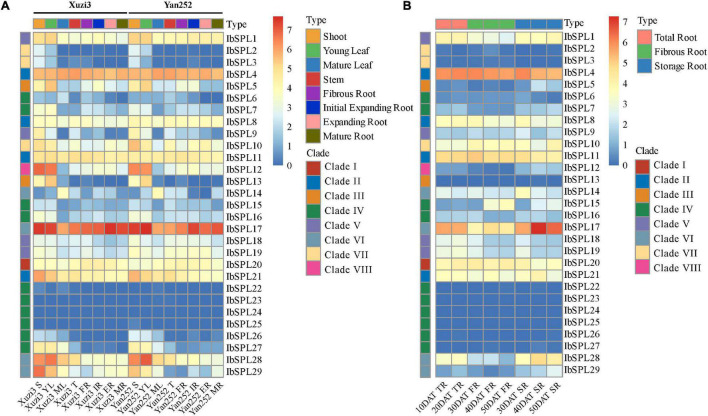
Expression profiles of *IbSPL* genes in different tissues and developmental stages using transcriptome analysis. **(A)** Heatmap representing the expression patterns of *IbSPL* genes in eight different tissues of two sweet potato cultivars (Xuzi3 and Yan252). Xuzi3: sweet potato cultivar Xuzi3, Yan252: sweet potato cultivar Yan252, S: shoot, YL: young leaf, ML: mature leaf, T: stem, FR: fibrous root, IR: initial tuberous root, ER: expanding storage root, MR: mature storage root. **(B)** Heatmap representing the expression patterns of *IbSPL* genes in eight different stages of root development. 10DAT_TR: undifferentiated root (10 days after transplanting), 20DAT_TR: undifferentiated root (20 days after transplanting), 30DAT_FR: fibrous root (30 days after transplanting), 40DAT_FR: fibrous root (days after transplanting), 50DAT_FR: fibrous root (50 days after transplanting), 30DAT_SR: storage root (30 days after transplanting), 40DAT_SR: storage root (40 days after transplanting), 50DAT_SR: storage root (50 days after transplanting). Heatmaps were generated based on the log_2_(FPKM + 1) values for each *IbSPL* gene.

Among the four *Ipomoea* species, *I. batatas* is the most important crop cultivated globally. To explore the putative roles of *IbSPL* genes, the tissue-specific expression patterns of *IbSPLs* were analyzed in eight tissues (four aboveground and four underground tissues) of two sweet potato cultivars (Xuzi3 and Yan252) using publicly available transcriptomic data (Supplementary Table 8; Ding et al., 2017). FPKM values were calculated to evaluate gene expression levels (Supplementary Table 9). As shown in Figure 5A, the expression patterns of *IbSPLs* were classified into three groups. The first group included seven *IbSPL* genes (*IbSPL6*/*IbSPL15*/*IbSPL22*/*IbSPL23*/*IbSPL24*/*IbSPL25*/*IbSPL26*), with lowest expression levels [log_2_(FPKM) < 2] in all tissues. The second group included five *IbSPL* genes (*IbSPL4*/*IbSPL8*/*IbSPL11*/*IbSPL17*/*IbSPL20*), with relatively high expression levels in all tissues. The third group included the remaining 17 *IbSPL* genes, with high expression in some aboveground tissues, especially in shoots or young leaves. Additionally, the gene expression profiles in underground tissues (fibrous and tuberous root) were investigated, with seven *IbSPL* genes highly expressed in underground tissues (mean FPKM > 10), such as *IbSPL1*, *IbSPL4*, *IbSPL11*, *IbSPL17*, *IbSPL20*, *IbSPL21*, and *IbSPL28*, implying their potential functionality in root development. Furthermore, these results showed that *IbSPL* genes within the same clades exhibit distinct expression patterns, such as *IbSPL14*, *IbSPL17*, *IbSPL28*, and *IbSPL29* in clade VI.

To further investigate the expression profiles of *IbSPL* genes in underground tissues, publicly available transcriptomic data of eight different stages during root development from the cultivar ‘Beauregard’ were used (Figure 5B and Supplementary Tables 8, 9). The results showed that the overall expression patterns of *IbSPL* genes in the roots of the cultivar ‘Beauregard’ were similar to those of cultivar ‘Xuzi3’ and ‘Yan252.’ Specifically, the expression levels of *IbSPL17*, *IbSPL28*, and *IbSPL29* were the highest in storage roots compared with undifferentiated and fibrous roots. *IbSPL1* showed the highest expression in undifferentiated roots whereas *IbSPL10* showed the highest expression in fibrous roots. However, the expression levels of *IbSPL4*, *IbSPL8*, *IbSPL11*, *IbSPL20*, and *IbSPL21* showed no distinct variation in all tissues. These results, therefore, imply that these genes may play important roles in root development.

**FIGURE 6:**
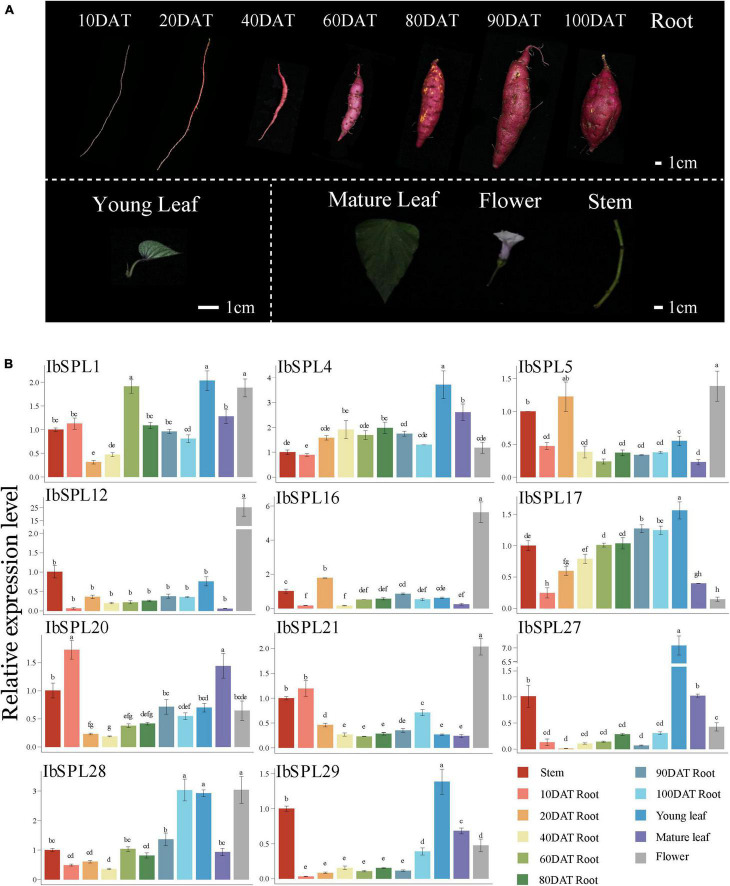
Expression profiles of 11 *IbSPL* genes in different tissues and at different developmental stages using qRT-PCR. **(A)** Different tissues from cultivar Xuyu34 are shown, including stem, leaf, flower, and root. Black bar is scale of 1 cm. **(B)** Expression patterns of 11 *IbSPL* genes determined using qRT-PCR. The *x*-axis indicates different tissues. The *y*-axis represents the relative expression calculated using the 2^–ΔΔ*CT*^ method. The error bars indicate the standard deviations of the three biological replicates. Different letters indicate statistically significant differences at *p* < 0.05.

To confirm the expression patterns of *IbSPLs* derived from the transcriptomic data, a total of 11 *IbSPL* genes (*IbSPL1*, *IbSPL4*, *IbSPL5*, *IbSPL12*, *IbSPL16*, *IbSPL17*, *IbSPL20*, *IbSPL21*, *IbSPL27*, *IbSPL28*, and *IbSPL29*) highly expressed in aboveground or underground tissues were selected for qRT-PCR analysis in 11 tissues of cultivar ‘Xuyu34’ (Figure 6). The results showed consistent expression patterns of *IbSPL* genes between the transcriptomic data and qRT-PCR results. Moreover, the expression levels of *IbSPLs* differed in various tissues. For example, *IbSPL27* and *IbSPL29* were highly expressed in young leaves, while *IbSPL12*, *IbSPL16*, and *IbSPL21* were highly expressed in flower. Moreover, the gradual increase in *IbSPL17* and *IbSPL28* expressions indicated their differential roles in storage root development in sweet potato.

### *IbSPL* Genes in Response to Exogenous Phytohormones

**FIGURE 7:**
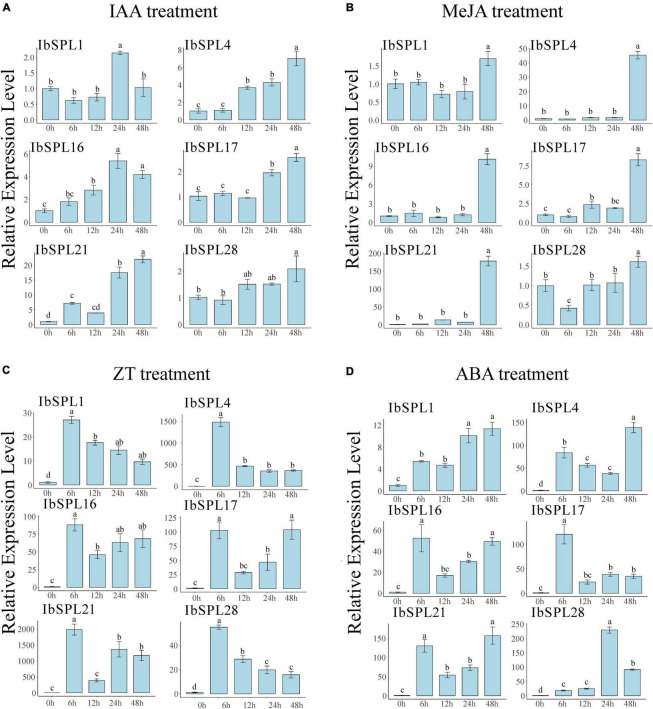
Expression patterns of six *IbSPL* genes under phytohormone treatment. **(A)** Expression patterns of the six *IbSPL* genes under IAA treatment. **(B)** Expression patterns of the six *IbSPL* genes under MeJA treatment. **(C)** Expression patterns of the six *IbSPL* genes under ZT treatment. **(D)** Expression patterns of the six *IbSPL* genes under ABA treatment. Samples were collected at 0, 6, 12, 24, and 48 h after treatment. The *x*-axis indicates different samples, while the *y*-axis represents the relative expression calculated using the 2^–ΔΔ*CT*^ method. The error bars indicate the standard deviations of the three biological replicates. Different letters indicate statistically significant differences at *p* < 0.05.

The promoter analysis revealed that *IbSPL* genes could be regulated by various phytohormones, which are known regulators of plant growth and development. To reveal the potential roles of *IbSPLs* in hormone signaling pathways, six *IbSPL* genes (*IbSPL1*, *IbSPL4*, *IbSPL16*, *IbSPL17*, *IbSPL21*, and *IbSPL28*) highly expressed in roots were selected to perform qRT-PCR analysis under exogenous phytohormone treatments, which included IAA, MeJA, ZT, and ABA. The expression analysis indicated that the six *IbSPL* genes exhibited highly divergent response patterns under phytohormone treatment in the adventitious root (Figure 7). Under IAA treatment, *IbSPL16* and *IbSPL21* were rapidly upregulated after 6 h of treatment, while the other four genes were upregulated after 12 or 24 h of treatment. Under MeJA treatment, all *IbSPL* genes were significantly upregulated after 48 h of treatment, with *IbSPL4* and *IbSPL21* upregulated around 45.3 and 178.1 folds, respectively. Under ZT treatment, all *IbSPL* genes were highly upregulated after 6 h of treatment, with a subsequent decline followed by approximately 10-fold increased expression than CK. Notably, *IbSPL21* showed a particularly positive response to ZT treatment. Under ABA treatment, all examined *IbSPL* genes, particularly *IbSPL1*, *IbSPL4*, *IbSPL16*, *IbSPL17*, and *IbSPL21*, were rapidly upregulated after 6 h of treatment, whereas *IbSPL28* showed significantly upregulation after 24 h of treatment.

### Regulatory Sub-Networks Involving *IbSPLs* and Other *I. batatas* Genes in the Storage Root of Sweet Potato

**FIGURE 8:**
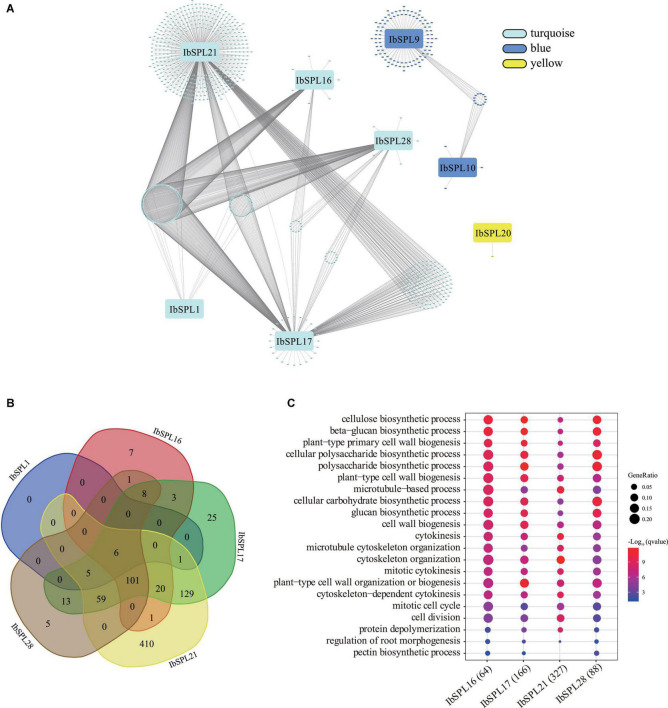
Regulatory sub-networks involving *IbSPLs* and other *I. batatas* genes in the storage root of sweet potato. **(A)** Co-expression sub-network visualization for *IbSPLs* in the storage root of sweet potato. Only the edges with a weight above a threshold of 0.05 are displayed. **(B)** Venn diagram of the number of co-expressed genes for *IbSPL1*, *IbSPL16, IbSPL17*, *IbSPL21*, and *IbSPL28* in the turquoise module. **(C)** GO enrichment analysis of co-expressed genes for *IbSPL16*, *IbSPL17*, *IbSPL21*, and *IbSPL28*.

To identify the regulatory sub-networks involving *IbSPLs* in the storage root of sweet potato, WGCNA was performed based on transcriptomic data of mature storage roots from 88 sweet potato accessions (Supplementary Figure 8 and Supplementary Table 11). A total of 19 modules were obtained from this analysis, with three modules (turquoise, blue and yellow) containing totally eight *IbSPL* genes (Figure 8A). In the yellow module, *IbSPL20* showed co-expression with only one gene. In the blue module, *IbSPL9* and *IbSPL10* were co-expressed with 17 and 107 genes, respectively. In the turquoise module, *IbSPL1*, *IbSPL16, IbSPL17*, *IbSPL21*, and *IbSPL28* had 12, 147, 370, 732, and 198 co-expressed genes, respectively (Supplementary Table 12). Interestingly, *IbSPL16, IbSPL17*, *IbSPL21*, and *IbSPL28* in the turquoise module share 101 co-expressed genes (Figure 8B), indicating functionality in similar biological processes.

To further explore the putative functions of the SPLs in the storage root, GO enrichment analysis was performed on the co-expressed genes. For *IbSPL1, IbSPL20*, and *IbSPL9*, GO enrichment results could not be obtained due to the small number of co-expressed genes. For *IbSPL10* in the blue module, co-expressed genes related to “response to chitin” and “response to organonitrogen compound” were enriched (Supplementary Table 13). For *IbSPL16*, *IbSPL17*, *IbSPL21*, and *IbSPL28* in the turquoise module, the similar GO terms were enriched, such as “regulation of root morphogenesis,” “cell division,” “cytoskeleton organization,” “plant-type cell wall organization or biogenesis,” and “cellulose biosynthetic process” (Figure 8C and Supplementary Table 13). It is known that the cytoskeleton, cell division, and cell wall organization/biogenesis are important biological processes involved in storage root development and formation (Dong et al., 2019). Therefore, these results indicated that *IbSPL16*/*IbSPL17*/*IbSPL21*/*IbSPL28* may play a key role in storage root development in sweet potato.

## Discussion

SPL genes are important plant-specific transcription factors with a highly conserved SBP domain. Since its discovery in *A. majus*, *SPL* gene members have been increasingly identified in plants (Klein et al., 1996; Cardon et al., 1999; Xie et al., 2006; Salinas et al., 2012; Li et al., 2013; Zhang et al., 2016; Tripathi et al., 2017). However, comprehensive molecular, evolutionary and functional analysis of the *SPL* genes in the genus *Ipomoea* are lacking. The genus *Ipomoea* has significant nutritional and economic value for humans, including the seventh most important crop *I. batatas* and ornamental plant *I. nil*. Up to now, four *Ipomoea* species have been sequenced: *I. nil*, *I. triloba*, *I. trifida*, and *I. batatas*. Utilizing these genomes, this study systematically analyzed the *Ipomoea SPL* genes, including molecular characteristics, evolutionary process, post-transcriptional regulation, and physiological function.

### Comparative Analysis of SQUAMOSA Promoter-Binding Protein-Like Genes in the Genus *Ipomoea*

Recently, some important transcription factor gene families have been investigated in *Ipomoea* species, such as bZIPs (Yang et al., 2019), WRKYs (Li Y. et al., 2019), GRPs (Lu et al., 2019), MADS (Zhu et al., 2020), and DEAD-box (Wan et al., 2020). However, these studies have focused only on the gene families in a single *Ipomoea* species, and comparative analysis of gene families in the genus *Ipomoea* are scarce. This study dissects the evolutionary dynamics of *SPL* genes in the genus *Ipomoea*, identifying *SPL* genes in four *Ipomoea* species: 29 *IbSPLs*, 27 *ItfSPLs*, 26 *ItbSPLs*, and 23 *IniSPLs* (Figure 1). Notably, the number of *SPL* genes in sweet potato is approximately equal to that of the diploid wild relatives (*I. nil*, *I. triloba*, and *I. trifida*), owing to the haplotype-resolved genome assembly of hexaploid sweet potato (Yang et al., 2017). Following the classifications of *A. thaliana, S. lycopersicum*, and *O. sativa* (Cardon et al., 1999; Xie et al., 2006; Salinas et al., 2012), the *Ipomoea SPL* genes were also divided into eight clades (Figure 2), with clade IV comprising the highest members (32) and clade I comprising the lowest (four). Gene and protein structure analysis revealed that most *Ipomoea* SPLs from the same phylogenetic clade share similar intron/exon structures, domain organizations, and motif compositions (Supplementary Figure 1), indicating that *SPLs* within the same clade may have similar functions in *Ipomoea* species. Interestingly, apart from the conserved domain and motifs present in the SPL proteins, other domains or motifs were found in clades I, II, and IV, such as DEXDc domain (motif 4) and Ankyrin repeats (motif 8), which were also observed in papaya (Xu et al., 2020) and barley (Tripathi et al., 2018). These results suggest that the SPLs in these clades may have undergone evolutionary functional differentiation and/or neofunctionalization.

*Ipomoea* species were found to possess more *SPL* genes than dicotyledonous model plants, such as *A. thaliana* and *S. lycopersicum* (Cardon et al., 1999; Salinas et al., 2012), implying the genus-specific expansion of the *SPL* gene family in *Ipomoea* species. The expansion of gene families is a result of evolutionary duplication events (Moore and Purugganan, 2005). This study showed that segmental duplication plays a major role in the evolution and expansion of *Ipomoea SPL* genes (Figure 3B), which is consistent with findings in cotton (Cai et al., 2018), *Rosacea* species (Abdullah et al., 2018) and *Euphorbiaceae* species (Li J. et al., 2019). Previous studies have reported that a whole-genome triplication event occurred 46.1 million years ago (Mya) in the progenitor of the genus *Ipomoea* (Yang et al., 2017), and thereby the derivation of the segmental duplication of SPLs from this event was speculated. Furthermore, tandem duplication was found to be the most frequent event in the IV-b and IV-c subclades (Supplementary Table 1) and orthologs were found to be absent in dicots (Supplementary Table 3), implying that the *SPL* members in these two subclades were tandem duplicated from a recent event. Therefore, the *SPL* genes were speculated to have undergone replication expansion in the progenitor of the genus *Ipomoea* and that various *SPL* members were retained due to their important role in growth and development during the *Ipomoea* species differentiation. Additionally, the *Ka/Ks* ratios were less than 1 for all *Ipomoea SPL* ortholog gene pairs, indicating that the *Ipomoea SPL* genes were under strong purifying selection (Supplementary Table 4).

Many *SPL* genes are miR156 targets, thus forming a functional regulatory network of miR156-SPL, which plays an important role in plant growth and development (Gou et al., 2011; Wang and Wang, 2015; Liu et al., 2019). More than half of the *SPL* gene family members have been reported to be targeted by miR156 in various plant species, such as rice (Xie et al., 2006), tomato (Salinas et al., 2012), and apple (Li et al., 2013). In this study, two-thirds of *SPL* genes in each *Ipomoea* species were predicted to be miR156 targets (Figure 4B). Phylogenetic analysis showed that all *SPL* genes in clade III lacked miR156 binding sites, which is consistent with the reported results of *A. thaliana*, rice, and tomato (Preston and Hileman, 2013). Additionally, two binding site types of miR156 were identified in *SPL* genes: one located in CDS and the other located in the 3′-UTR (Figure 4B), which is consistent with the observation in other plants, such as rice (Xie et al., 2006), tomato (Salinas et al., 2012), apple (Li et al., 2013), and papaya (Xu et al., 2020). The degradome data of *I. batatas* further confirmed seven *IbSPL* genes as the targets of miR156 (Figure 4C and Supplementary Table 1). Among these *IbSPL* genes, the miR156 binding site for *IbSPL17* and *IbSPL28* is located in the 3′-UTR, which is consistent with the miR156 binding site of their orthologous genes (*AtSPL3* and *LeSPL-CNR*) in *A. thaliana* and tomato (Gandikota et al., 2007; Chen et al., 2015). This suggests the high conservation of the miR156-SPL regulatory module in plants. Additionally, our results showed a negative correlation in the expression pattern of miR156 and their target *SPL* genes, suggesting that *SPLs* might be regulated by miR156 at the post-transcriptional level. However, most of the miR156-SPL interactions in this study were predicted using *in silico* analysis, requiring further experimental verification for the miR156-SPL interactions in the genus *Ipomoea*.

### *IbSPL* Genes Are Putatively Involved in Storage Root Development

Sweet potato, the seventh most important crop globally, has strong adaptability, stable yield, and high nutritional value (Liu, 2017). The storage root of sweet potato is economically useful for its nutrient content and yield, and thus, dissecting the mechanisms underlying storage root formation and development is significant to improve sweet potato nutrient content and yield. Considering the key regulatory roles of *SPL* genes in root architecture (Yu et al., 2015; Barrera-Rojas et al., 2020) and biomass enhancement (Wang et al., 2015), the expression patterns of *IbSPL* genes in different tissues or at different developmental stages were evaluated using the public transcriptome data. Most of the *IbSPLs* were found to be highly expressed in aboveground tissues, especially in shoots or young leaves; however, only some *IbSPLs* were found to be highly expressed in underground tissues (Figure 7). qRT-PCR analysis of the expression levels of two *IbSPL* genes (*IbSPL17*/*IbSPL28*) revealed a significant increase with storage root development (Figure 6). This study provides evidence that *SPL* genes have important functions during storage root development in sweet potato.

The formation and development of storage roots is a complex physiological process that includes the cessation of root elongation, genesis and development of the primary and secondary vascular cambium, increase in radial growth and accumulation of starch and storage proteins (Ravi et al., 2009). These processes are closely related to the endogenous phytohormones, such as IAA, cytokinins (CTKs), JA, and ABA (Nakatani, 1991; Tanaka et al., 2008; Ravi et al., 2009; Dong et al., 2019). For instance, IAA is involved in early stages of storage root formation and primary storage root thickening (Noh et al., 2010); ABA plays a significant role in storage root bulking by activating cell division (Huan et al., 2020) and CTKs play a key role in storage root initiation and expansion as a pre-requirement for cambial cell proliferation (Dong et al., 2019). Moreover, storage root yields are positively correlated with ABA and CTK contents (Wang et al., 2005). In the present study, different kinds of hormone-responsive elements were found by analyzing the *IbSPL* promoters (Supplementary Figure 7), implying that *IbSPL* genes may participate in hormone signaling pathways. qRT-PCR analysis further confirmed that the expression of the tested *IbSPLs* (*IbSPL1*, *IbSPL4*, *IbSPL16*, *IbSPL17*, *IbSPL21*, and *IbSPL28*) was strongly induced under exogenous phytohormone treatments, particularly ZT and ABA, suggesting their crucial roles in root development.

Storage root formation and development is maintained by coordinated cellular behaviors, such as cell division, expansion, and differentiation. Previous studies have revealed that cell wall biosynthesis and cytoskeleton organization are critical in these cellular behaviors (Bashline et al., 2014; Brasil et al., 2017). The regulatory sub-networks in this study were analyzed using WGCNA, which indicated that eight *IbSPL* genes were co-expressed with at least one other *I. batatas* genes (Figure 8A). GO enrichment analysis of co-expressed genes speculated the role of *IbSPL* genes in stress responses, root morphogenesis, and cell division (Supplementary Table 13). Moreover, the genes co-expressed with *IbSPL16*/*IbSPL17*/*IbSPL21*/*IbSPL28* in the turquoise module were all significantly enriched for “regulation of root morphogenesis,” “cell division,” “cytoskeleton organization,” “plant-type cell wall organization or biogenesis,” and “cellulose biosynthetic process.” These enriched processes are essential for cell morphogenesis and cell cycles, implying their key roles in storage root development. In the future, functional characterization is needed to elucidate the specific roles of *IbSPLs* in storage root development.

## Conclusion

In summary, a genome-wide analysis of the *SPL* gene family in four *Ipomoea* species, including *I. batatas*, *I. trifida*, *I. triloba*, and *I. nil* was performed. A total of 105 *Ipomoea SPL* genes were identified and divided into eight clades. Genes in one clade were found to harbor similar gene structures, domain organizations, motif compositions, and *cis*-acting elements, suggesting potential functional similarity. Moreover, segmental duplication was predominantly responsible for the expansion of the *Ipomoea SPL* gene family. On combining the results from the expression patterns and regulatory sub-networks, *IbSPL16*/*IbSPL17*/*IbSPL21*/*IbSPL28* were found to play an important role in storage root development. Therefore, this study not only provides novel insights into the evolutionary and functional divergence of the *SPL* genes in the genus *Ipomoea* but also lays a foundation for further elucidation of the potential functional roles of *IbSPL* genes during storage root development.

## Data Availability Statement

The datasets presented in this study can be found in online repositories. The names of the repository/repositories and accession number(s) can be found in the article/Supplementary Material.

## Author Contributions

LZ, SW, and TX conceived and designed the research. HS, JM, WZ, LZ, WH, and YZ performed the research and analyzed the data. LZ and HS wrote the manuscript. All authors have read and approved the manuscript.

## Conflict of Interest

The authors declare that the research was conducted in the absence of any commercial or financial relationships that could be construed as a potential conflict of interest.

## Publisher’s Note

All claims expressed in this article are solely those of the authors and do not necessarily represent those of their affiliated organizations, or those of the publisher, the editors and the reviewers. Any product that may be evaluated in this article, or claim that may be made by its manufacturer, is not guaranteed or endorsed by the publisher.

## Footnotes

1
http://viewer.shigen.info/asagao

2
http://sweetpotato.uga.edu

3
http://public-genomes-ngs.molgen.mpg.de/SweetPotato

4
https://www.arabidopsis.org
