# Supplementary material for: Phylogenetic Analysis of the SQUAMOSA Promoter-Binding Protein-Like Genes in Four Ipomoea Species and Expression Profiling of the IbSPLs During Storage Root Development in Sweet Potato (Ipomoea batatas)
Source: Front Plant Sci. 2022 Jan 21;12:801061. doi: 10.3389/fpls.2021.801061 (PMC8815303; doi:10.3389/fpls.2021.801061)

a

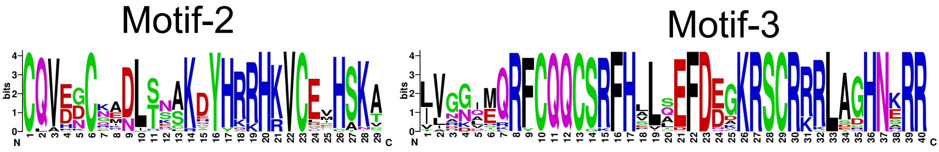

b

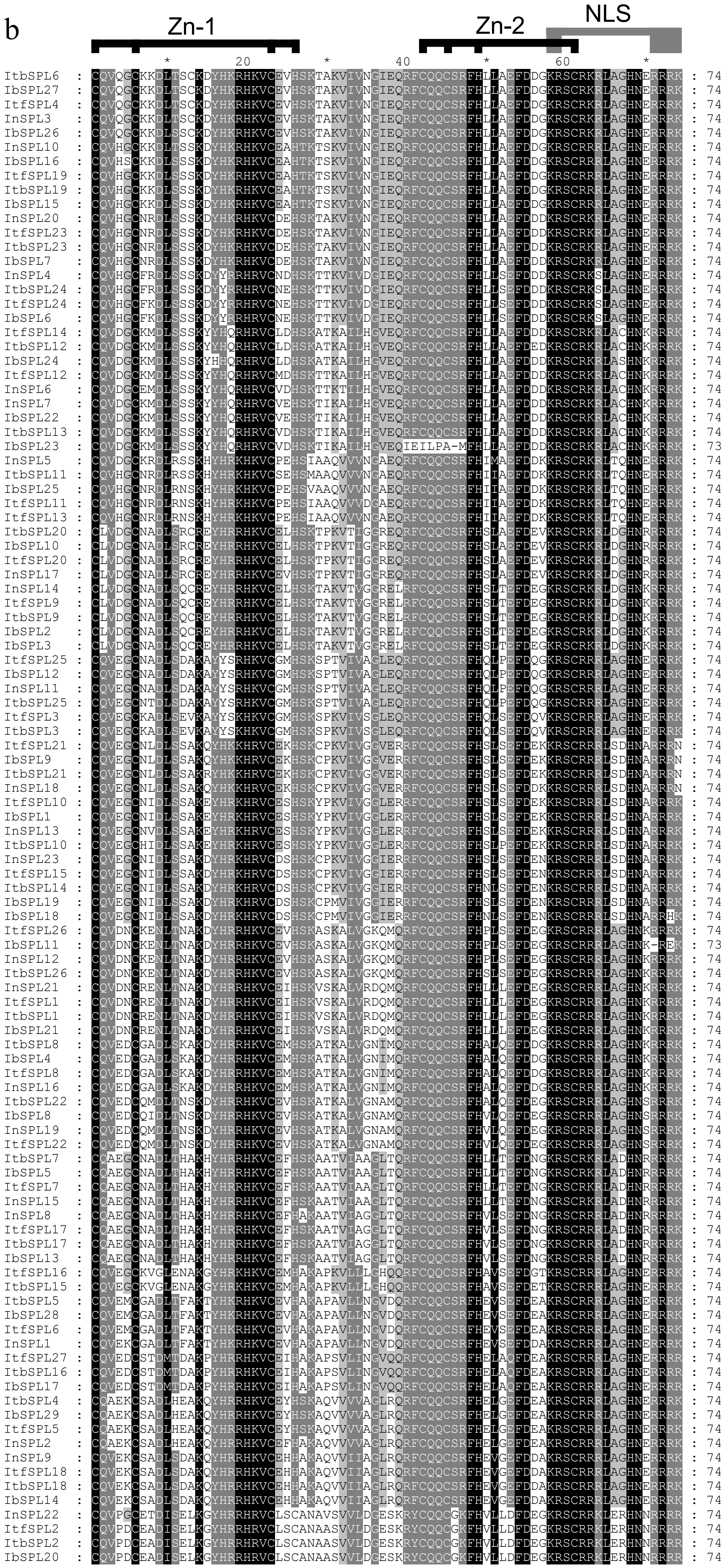

Supplement: Supplementary file 1 [file Data_Sheet_1.zip › Suplementary_materials/Supplementary Figure S2.pdf]
