# Supplementary material for: Phylogenetic Analysis of the SQUAMOSA Promoter-Binding Protein-Like Genes in Four Ipomoea Species and Expression Profiling of the IbSPLs During Storage Root Development in Sweet Potato (Ipomoea batatas)
Source: Front Plant Sci. 2022 Jan 21;12:801061. doi: 10.3389/fpls.2021.801061 (PMC8815303; doi:10.3389/fpls.2021.801061)
Supplement: Supplementary file 1 [file Data_Sheet_1.zip › Suplementary_materials/Supplementary Figure S3.pdf]

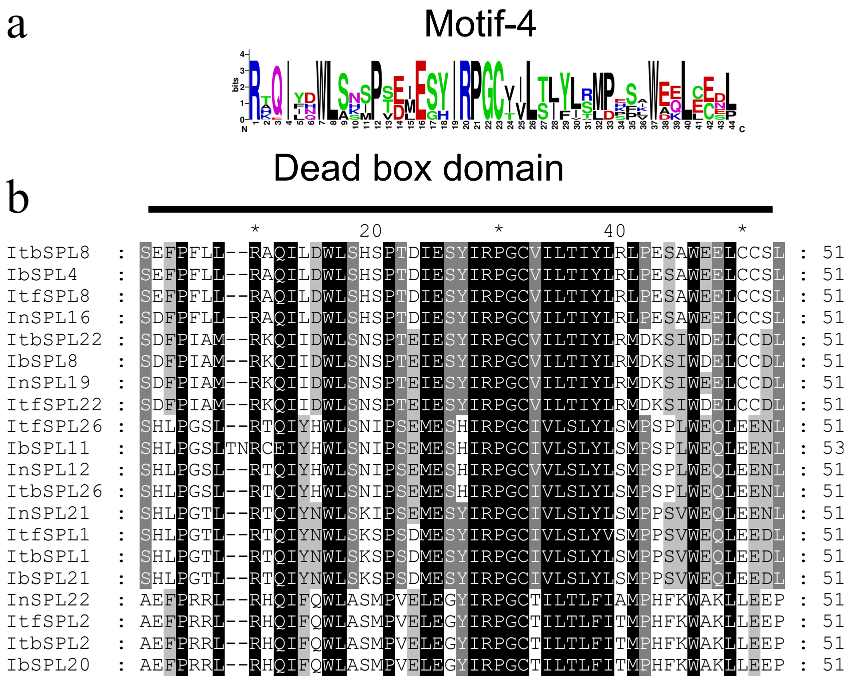
a
Motif-4
bits
b
Dead box domain
* 20 * 40 *
ItbSPL8 : SEFPFLL--RAQILDWLSHSPTDIESYIRPGCVILTIYLRLPESAWEELCCSL : 51
IbSPL4 : SEFPFLL--RAQILDWLSHSPTDIESYIRPGCVILTIYLRLPESAWEELCCSL : 51
ItfSPL8 : SEFPFLL--RAQILDWLSHSPTDIESYIRPGCVILTIYLRLPESAWEELCCSL : 51
InSPL16 : SDFPFLL--RAQILDWLSHSPTDIESYIRPGCVILTIYLRLPESAWEELCCSL : 51
ItbSPL22 : SDFPIAM--RKQIIDWLSNSPTEIESYIRPGCVILTIYLRMDKSIWDELCCDL : 51
IbSPL8 : SDFPIAM--RKQIIDWLSNSPTEIESYIRPGCVILTIYLRMDKSIWDELCCDL : 51
InSPL19 : SDFPIAM--RKQIIDWLSNSPTEIESYIRPGCVILTIYLRMDKSIWEELCCDL : 51
ItfSPL22 : SDFPIAM--RKQIIDWLSNSPTEIESYIRPGCVILTIYLRMDKSIWDELCCDL : 51
ItfSPL26 : SHLPGSL--RTQIYHWLSNIPSEMESHIRPGCIVLSLYLSMPSPLWEQLEENL : 51
IbSPL11 : SHLPGSLTNRCEIYHWLSNIPSEMESHIRPGCIVLSLYLSMPSPLWEQLEENL : 53
InSPL12 : SHLPGSL--RTQIYHWLSNIPSEMESHIRPGCVVLSLYLSMPSPLWEQLEENL : 51
ItbSPL26 : SHLPGSL--RTQIYHWLSNIPSEMESHIRPGCIVLSLYLSMPSPLWEQLEENL : 51
InSPL21 : SHLPGTL--RTQIYNWLSKIPSEMESYIRPGCIVLSLYLSMPPSVWEQLEENL : 51
ItfSPL1 : SHLPGTL--RTQIYNWLSKSPSDMESYIRPGCIVLSLYVSMPPSVWEQLEEDL : 51
ItbSPL1 : SHLPGTL--RTQIYNWLSKSPSDMESYIRPGCIVLSLYLSMPPSVWEQLEEDL : 51
IbSPL21 : SHLPGTL--RTQIYNWLSKSPSDMESYIRPGCIVLSLYLSMPPSVWEQLEEDL : 51
InSPL22 : AEFPRRL--RHQIFQWLASMPVELEGYIRPGCTILTLFIAMPHFKWAKLLEEP : 51
ItfSPL2 : AEFPRRL--RHQIFQWLASMPVELEGYIRPGCTILTLFITMPHFKWAKLLEEP : 51
ItbSPL2 : AEFPRRL--RHQIFQWLASMPVELEGYIRPGCTILTLFITMPHFKWAKLLEEP : 51
IbSPL20 : AEFPRRL--RHQIFQWLASMPVELEGYIRPGCTILTLFITMPHFKWAKLLEEP : 51
