# Supplementary material for: Phylogenetic Analysis of the SQUAMOSA Promoter-Binding Protein-Like Genes in Four Ipomoea Species and Expression Profiling of the IbSPLs During Storage Root Development in Sweet Potato (Ipomoea batatas)
Source: Front Plant Sci. 2022 Jan 21;12:801061. doi: 10.3389/fpls.2021.801061 (PMC8815303; doi:10.3389/fpls.2021.801061)
Supplement: Supplementary file 1 [file Data_Sheet_1.zip › Suplementary_materials/Supplementary Figure S4.pdf]

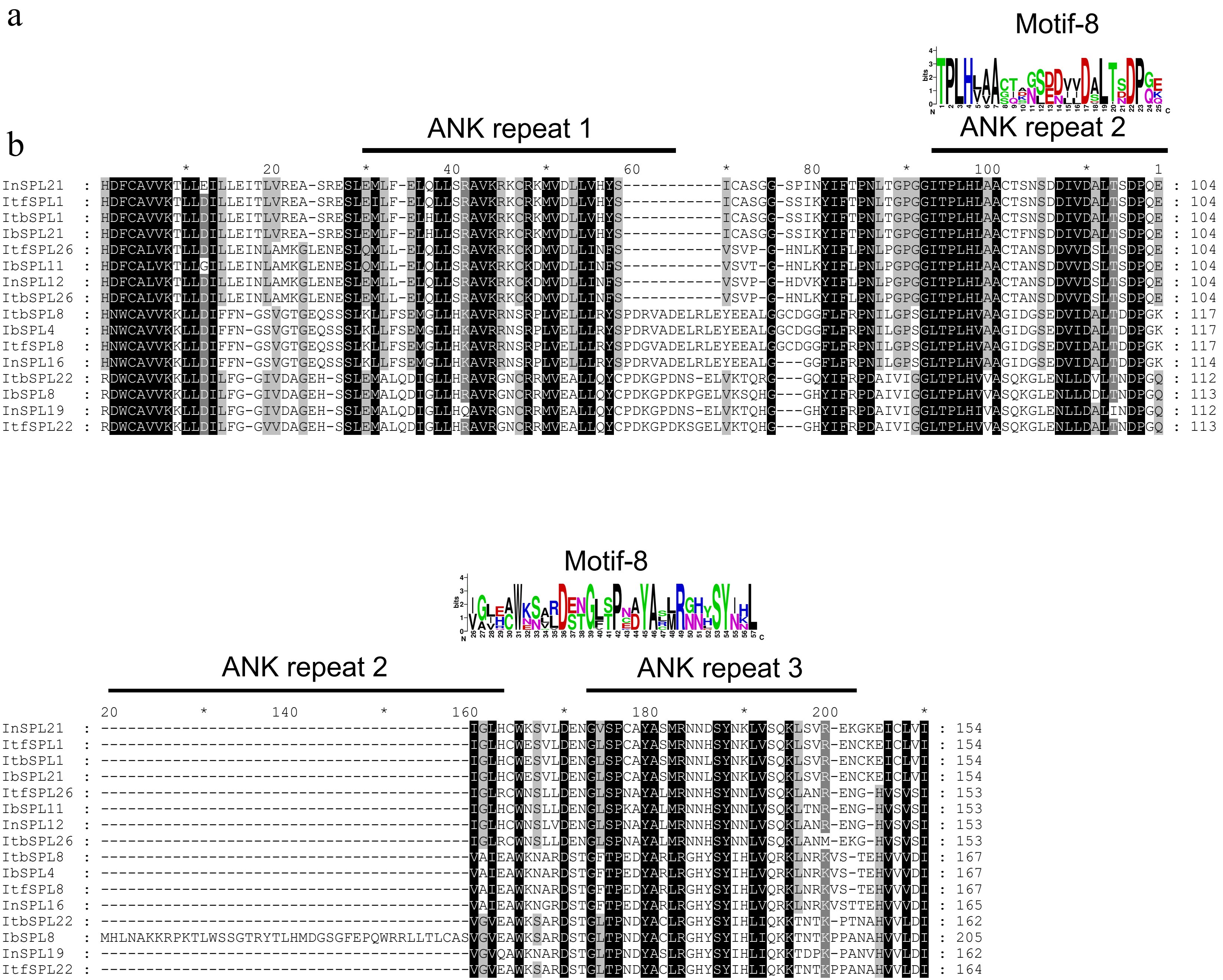
a
Motif-8
b
ANK repeat 1
ANK repeat 2
* 20 * 40 * 60 * 80 * 100 * 1
InSPL21 : HDFCAVVKTLLEILLEITLVREA-SRESLEMLF-ELQLLSRAVKRKCRKMVDLLVHYS-----------ICASGG-SPINYIFTPNLTGPGGITPLHIAACTSNSDDIVDALTSDPQE : 104
ItfSPL1 : HDFCAVVKTLLDILLEITLVREA-SRESLEILF-ELQLLSRAVKRKCRKMVDLLVHYS-----------ICASGG-SSIKYIFTPNLTGPGGITPLHIAACTSNSDDIVDALTSDPQE : 104
ItbSPL1 : HDFCAVVKTLLDILLEITLVREA-SRESLEMLF-ELHLLSRAVKRKCRKMVDLLVHYS-----------ICASGG-SSIKYIFTPNLTGPGGITPLHIAACTSNSDDIVDALTSDPQE : 104
IbSPL21 : HDFCAVVKTLLDILLEITLVREA-SRESLEMLF-ELHLLSRAVKRKCRKMVDLLVHYS-----------ICASGG-SSIKYIFTPNLTGPGGITPLHIAACTFNSDDIVDALTSDPQE : 104
ItfSPL26 : HDFCALVKTLLDILLEINLAMKGLENESLQMLL-ELQLLSRAVKRKCKDMVDLLINFS-----------VSVP-G-HNLKYIFLPNLPGPGGITPLHIAACTANSDDVVDSLTSDPQE : 104
IbSPL11 : HDFCALVKTLLGILLEINLAMKGLENESLQMLL-ELQLLSRAVKRKCKDMVDLLINFS-----------VSVT-G-HNLKYIFLPNLPGPGGITPLHIAACTANSDDVVDSLTSDPQE : 104
InSPL12 : HDFCALVKTLLDILLEINLAMKGLENESLEMLL-ELQLLSRAVKRKCKDMVDLLINFS-----------VSVP-G-HDVKYIFLPNLPGPGGITPLHIAACTANSDDVVDSLTSDPQE : 104
ItbSPL26 : HDFCALVKTLLDILLEINLAMKGLENESLEMLL-ELQLLSRAVKRKCKDMVDLLINFS-----------VSVP-G-HNLKYIFLPNLPGPGGITPLHIAACTANSDDVVDSLTSDPQE : 104
ItbSPL8 : HNWCAVVKKLLDIFFN-GSVGTGEQSSSLKLLFSEMGLLHKAVRRNSRPLVELLLRYSPDRVADELRLEYEEALGGCDGGFLFRPNILGPSGLTPLHVAAGIDGSEDVIDALTDDPGK : 117
IbSPL4 : HNWCAVVKKLLDIFFN-GSVGTGEQSSSLKLLFSEMGLLHKAVRRNSRPLVELLLRYSPDRVADELRLEYEEALGGCDGGFLFRPNILGPSGLTPLHVAAGIDGSEDVIDALTDDPGK : 117
ItfSPL8 : HNWCAVVKKLLDIFFN-GSVGTGEQSSSLKLLFSEMGLLHKAVRRNSRPLVELLLRYSPDGVADELRLEYEEALGGCDGGFLFRPNILGPSGLTPLHVAAGIDGSEDVIDALTDDPGK : 117
InSPL16 : HNWCAVVKKLLDIFFN-GSVGTGEQSSSLKLLFSEMGLLHKAVRRNSRPLVELLLRYSPDRVADELRLEYEEALG---GGFLFRPNILGPSGLTPLHVAAGIDGSEDVIDALTDDPGK : 114
ItbSPL22 : RDWCAVVKKLLDILFG-GIVDAGEH-SSLEMALQDIGLLHRAVRGNCRRMVEALLQYCPDKGPDNS-ELVKTQRG---GQYIFRPDAIVIGGLTPLHVVASQKGLENLLDVLTNDPGQ : 112
IbSPL8 : RDWCAVVKKLLDILFG-GIVDAGEH-SSLEMALQDIGLLHRAVRGNCRRMVEALLQYCPDKGPDKPGELVKSQHG---GHYIFRPDAIVIGGLTPLHVVASQKGLENLLDDLTNDPGQ : 113
InSPL19 : RDWCAVVKKLLDILFG-GIVDAGEH-SSLEMALQDIGLLHQAVRGNCRRMVEALLQYCPDKGPDNS-ELVKTQHG---GHYIFRPDAIVIGGLTPLHIVASQKGLENLLDALINDPGQ : 112
ItfSPL22 : RDWCAVVKKLLDILFG-GVVDAGEH-SSLEMALQDIGLLHRAVRGNCRRMVEALLQYCPDKGPDKSGELVKTQHG---GHYIFRPDAIVIGGLTPLHVVASQKGLENLLDALTNDPGQ : 113
Motif-8
ANK repeat 2
ANK repeat 3
20 * 140 * 160 * 180 * 200 *
InSPL21 : --IGLHCWKSVLDENGVSPCAYASMRNNDSYNKLVSQKLSVR-EKGKEICLVI : 154
ItfSPL1 : --IGLHCWESVLDENGLSPCAYASMRNNHSYNKLVSQKLSVR-ENCKEICLVI : 154
ItbSPL1 : --IGLHCWESVLDENGLSPCAYASMRNNLSYNKLVSQKLSVR-ENCKEICLVI : 154
IbSPL21 : --IGLHCWESVLDENGLSPCAYASMRNNLSYNKLVSQKLSVR-ENCKEICLVI : 154
ItfSPL26 : --IGLRCWNSLLDENGLSPNAYALMRNNHSYNNLVSQKLANR-ENG-HVSVSI : 153
IbSPL11 : --IGLHCWNSLLDENGLSPKAYALMRNNHSYNNLVSQKLTNR-ENG-HVSVSI : 153
InSPL12 : --IGLHCWNSLVDENGLSPNAYALMRNNHSYNNLVSQKLANR-ENG-HVSVSI : 153
ItbSPL26 : --IGLRCWNSLLDENGLSPNAYALMRNNHSYNNLVSQKLANM-EKG-HVSVSI : 153
ItbSPL8 : --VAIEAWKNARDSTGFTPEDYARLRGHYSYIHLVQRKLNRKVS-TEHVVVDI : 167
IbSPL4 : --VAIEAWKNARDSTGFTPEDYARLRGHYSYIHLVQRKLNRKVS-TEHVVVDI : 167
ItfSPL8 : --VAIEAWKNARDSTGFTPEDYARLRGHYSYIHLVQRKLNRKVS-TEHVVVDI : 167
InSPL16 : --VAIEAWKNGRDSTGFTPEDYARLRGHYSYIHLVQRKLNRKVSTTEHVVVDI : 165
ItbSPL22 : --VGVEAWKSARDSTGLTPNDYACLRGHYSYIHLIQKKTNTK-PTNAHVVLDI : 162
IbSPL8 : MHLNAKKRPKTLWSSGTRYTLHMDGSGFEPQWRRLLTLCASVGVEAWKSARDSTGLTPNDYACLRGHYSYIHLIQKKTNTKPPANAHVVLDI : 205
InSPL19 : --VGVQAWKNARDSTGLTPNDYASLRGHYSYIHLIQKKTDPK-PANVHVVLDI : 162
ItfSPL22 : --VGVEAWKSARDSTGLTPNDYACLRGHYSYIHLIQKKTNTKPPANAHVVLDI : 164
