# Supplementary material for: Phylogenetic Analysis of the SQUAMOSA Promoter-Binding Protein-Like Genes in Four Ipomoea Species and Expression Profiling of the IbSPLs During Storage Root Development in Sweet Potato (Ipomoea batatas)
Source: Front Plant Sci. 2022 Jan 21;12:801061. doi: 10.3389/fpls.2021.801061 (PMC8815303; doi:10.3389/fpls.2021.801061)
Supplement: Supplementary file 1 [file Data_Sheet_1.zip › Suplementary_materials/Supplementary_Material.docx]

Supplementary Material

# Supplementary Figures

**Supplementary Figure S1.** Phylogenetic relationships, gene structures, domain organizations, and motif compositions of *SPL* genes in *Ipomoea* species

(a) The phylogenetic tree of 105 *Ipomoea* SPL proteins. (b) The intron-exon structures of *Ipomoea* *SPL* genes, wherein the red box indicates coding sequence and the black line represents intron. (c) The domain organization of *Ipomoea* SPL proteins. SBP domain, ANK repeat and DEXDc domain are represented by blue, purple, and orange boxes, respectively. (d) The motif compositions of *Ipomoea* SPL proteins. The ten motifs are indicated by different colored boxes. The length of a gene or protein can be estimated using the scale at the bottom.

**Supplementary Figure S2.** Conserved SBP domain and motifs in *Ipomoea* SPL proteins

(a) Sequence logo of the conserved motifs in SBP domain. The motifs were identified by the MEME software. The height of each stack indicates the degree of conservation at each position, and the height of the letters within each stack represents the relative frequency of the corresponding amino acid. (b) Multiple alignment of the conserved SBP domain. The two non-interleaved zinc finger-like structures (Zn-1/2) and one nuclear localization signal (NLS) were indicated on the top. Multiple sequence alignment of the SBP domain were shown on the below.

**Supplementary Figure S3.** Conserved DEXDc domain and motifs in *Ipomoea* SPL proteins

(a) Sequence logo of the conserved motifs in DEXDc domain. The motifs were identified by the MEME software. The height of each stack indicates the degree of conservation at each position, and the height of the letters within each stack represents the relative frequency of the corresponding amino acid. (b) Multiple alignment of the conserved DEXDc domain.

**Supplementary Figure S4.** Conserved ANK repeats and motifs in *Ipomoea* SPL proteins

(a) Sequence logo of the conserved motifs in ANK repeats. The motifs were identified by the MEME software. The height of each stack indicates the degree of conservation at each position, and the height of the letters within each stack represents the relative frequency of the corresponding amino acid. (b) Multiple alignment of the conserved ANK repeats.

**Supplementary Figure S5.** Sequence logo of the ten conserved motifs

The height of each stack indicates the degree of conservation at each position, and the height of the letters within each stack represents the relative frequency of the corresponding amino acid.

**Supplementary Figure S6.** Syntenic relationships of *Ipomoea* SPLs

(a) The colinear relationship among *SPL* genes in *I. trifida*. (b) The colinear relationship among *SPL* genes in *I. triloba*. (c) The colinear relationship among *SPL* genes in *I. nil*. Colored lines indicate syntenic regions. *Ipomoea SPL* genes can be classified into four duplicated types, and marked as different colors.

Supplementary Figure S7. Investigation of *cis*-acting elements in the *Ipomoea* *SPL* genes

(a) *Cis*-acting elements in the promoter regions of the *Ipomoea SPL* genes. (b) Pie charts of the *cis*-acting elements in the *SPL* genes, wherein the colored boxes represent different categories or *cis*-acting elements.

**Supplementary Figure S8.** WGCNA network and module detection

(a) Sample clustering was performed to detect outliers. CRR022914, CRR022943, and CRR022951 were considered as outlier samples, and were removed from the further analysis. (b, c) Selection of the soft-thresholding powers. Power 9 was selected, for which the fit index curve flattens out upon reaching a high value. (d) Cluster dendrogram and module assignment. Each color indicates one assigned module, and branches represent genes. 19 modules with 19431 genes were detected. (e) Heatmap shows the Topological Overlap Matrix (TOM) of 1000 genes selected randomly for WGCNA. (f) The cluster dendrogram constructed based on the eigengenes of the modules (excluding grey module) (above) and the heatmap for the correlation coefficient between the modules (below).

# Supplementary Tables

**Supplementary Table S1.** Summary of *SPL* family members in genus *Ipomoea*

**Supplementary Table S2.** List of *SPL* genes from *A. thaliana*, *O. sativa*, *S. lycopersicum*, *P. trichocarpa*, *M. domestica*, *J. curcas* and *C. reinhardii*

**Supplementary Table S3.** The orthologous groups among *O. sativa, A. thaliana, S. lycopersicum* and the four *Ipomoea* species

**Supplementary Table S4.** The Ka, Ks and Ka/Ks ratio for all orthologous groups

**Supplementary Table S5.** List of transcriptomic data that were used for miR156 target site prediction

**Supplementary Table S6.** The composition of cis-regulatory elements in promoter region of *SPL* genes among four *Ipomoea* species

**Supplementary Table S7.** The cis-regulatory elements identified in more than ten *Ipomoea* *SPL* genes

**Supplementary Table S8.** List of transcriptomic data that were used for expression analysis

**Supplementary Table S9.** The FPKM values for *IbSPL* genes in different tissues

**Supplementary Table S10.** Primers used for qRT-PCR

**Supplementary Table S11.** List of transcriptomic data that were used for co-expression sub-network construction

**Supplementary Table S12.** List of genes co-expressed with *IbSPLs*

**Supplementary Table S13.** GO enrichment analysis of genes co-expressed with *IbSPLs*
