# Supplementary material for: Phylogenetic Analysis of the SQUAMOSA Promoter-Binding Protein-Like Genes in Four Ipomoea Species and Expression Profiling of the IbSPLs During Storage Root Development in Sweet Potato (Ipomoea batatas)
Source: Front Plant Sci. 2022 Jan 21;12:801061. doi: 10.3389/fpls.2021.801061 (PMC8815303; doi:10.3389/fpls.2021.801061)
Supplement: Supplementary file 1 [file Supplementary Figure S6.pdf › Suplementary_materials/Supplementary Figure S6.pdf]

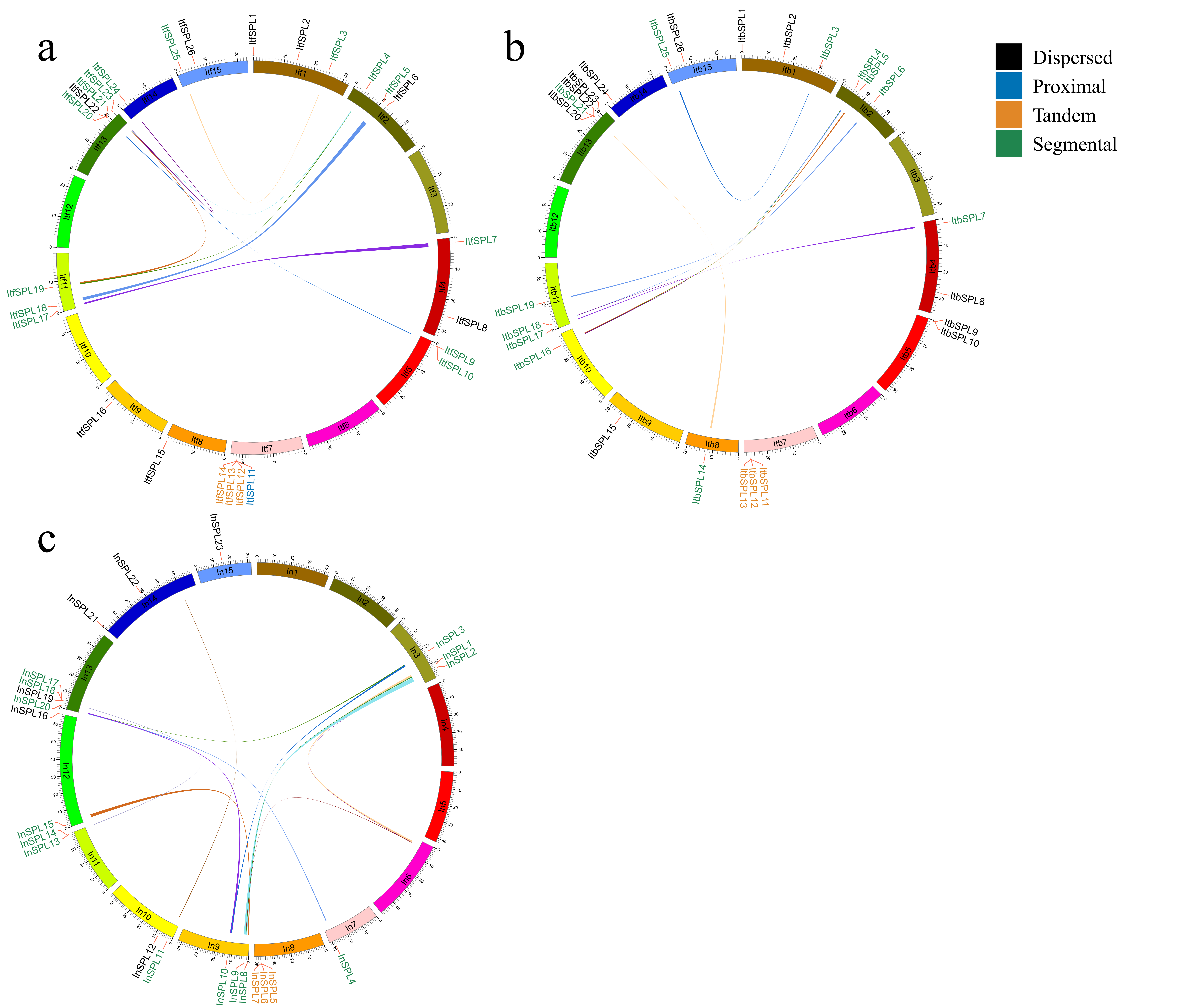

a
b
c
Dispersed
Proximal
Tandem
Segmental
ItfSPL1
ItfSPL2
ItfSPL3
ItfSPL4
ItfSPL5
ItfSPL6
ItfSPL7
ItfSPL8
ItfSPL9
ItfSPL10
ItfSPL11
ItfSPL12
ItfSPL13
ItfSPL14
ItfSPL15
ItfSPL16
ItfSPL17
ItfSPL18
ItfSPL19
ItfSPL20
ItfSPL21
ItfSPL22
ItfSPL23
ItfSPL24
ItfSPL25
ItfSPL26
ItbSPL1
ItbSPL2
ItbSPL3
ItbSPL4
ItbSPL5
ItbSPL6
ItbSPL7
ItbSPL8
ItbSPL9
ItbSPL10
ItbSPL11
ItbSPL12
ItbSPL13
ItbSPL14
ItbSPL15
ItbSPL16
ItbSPL17
ItbSPL18
ItbSPL19
ItbSPL20
ItbSPL21
ItbSPL22
ItbSPL23
ItbSPL24
ItbSPL25
ItbSPL26
InSPL1
InSPL2
InSPL3
InSPL4
InSPL5
InSPL6
InSPL7
InSPL8
InSPL9
InSPL10
InSPL11
InSPL12
InSPL13
InSPL14
InSPL15
InSPL16
InSPL17
InSPL18
InSPL19
InSPL20
InSPL21
InSPL22
InSPL23
